# ﻿*Pulvinatusia* (Brassicaceae), a new cushion genus from China and its systematic position

**DOI:** 10.3897/phytokeys.189.77926

**Published:** 2022-01-24

**Authors:** Hong-Liang Chen, Ihsan A. Al-Shehbaz, Li-Shen Qian, Jian-Wen Zhang, Bo Xu, Ti-Cao Zhang, Ji-Pei Yue, Hang Sun

**Affiliations:** 1 CAS Key Laboratory for Plant Diversity and Biogeography of East Asia, Kunming Institute of Botany, Chinese Academy of Sciences, Kunming 650201, Yunnan, China Kunming Institute of Botany, Chinese Academy of Sciences Kunming China; 2 Laboratory of Systematics & Evolutionary Botany and Biodiversity, College of Life Science, Zhejiang University, Hangzhou 310058, Zhejiang, China Zhejiang University Hangzhou China; 3 Missouri Botanical Garden, 4344 Shaw Boulevard, St. Louis, Missouri 63110, USA Missouri Botanical Garden St. Louis United States of America; 4 University of Chinese Academy of Sciences, Beijing 100049, China University of Chinese Academy of Sciences Beijing China; 5 College of Forestry, Southwest Forestry University, Kunming 650224, Yunnan, China Southwest Forestry University Kunming China; 6 College of Chinese Material Medica, Yunnan University of Chinese Medicine, Kunming 650500, Yunnan, China Yunnan University of Chinese Medicine Kunming China

**Keywords:** Crucihimalayeae, cushion plants, molecular phylogenetics, new species, Xizang

## Abstract

The new genus and species *Pulvinatusiaxuegulaensis* (Brassicaceae) are described and illustrated. The species is a cushion plant collected from Xuegu La, Xizang, China. Its vegetative parts are most similar to those of *Arenariabryophylla* (Caryophyllaceae) co-occurring in the same region, while its leaves and fruits closely resemble those of *Xerodrabapatagonica* (Brassicaceae) from Patagonian Argentina and Chile. Family-level phylogenetic analyses based on both nuclear ITS and plastome revealed that it is a member of the tribe Crucihimalayeae, but the infra-/intergeneric relationships within the tribe are yet to be resolved.

## ﻿Introduction

Cushion plants represent a special life form which usually has character combinations such as short-node intervals, compact branches, solitary flowers or few-flowered racemes, and dome- or mat-shaped cushions. They are common among perennial herbs growing on high-altitude mountains and are thought to be associated with dry and cold environments, such as the high Andes and Patagonia, Himalayas, and New Zealand Alps (Aubert et al. 2014; Boucher et al. 2016). Hauri and Schröter (1914) compiled the first worldwide list of cushion plants which included 338 species of 34 families and 78 genera. A century later, Aubert et al. (2014) updated the cushion plants catalogue in which they recognized 1,309 species of 63 families and 273 genera. An online database was also created for easy access and timely update (http://www.cushionplants.eu/).

The mustard family (Brassicaceae) is distributed primarily in temperate areas, and many of its species grow on high mountains. Aubert et al. (2014) reported 100 species from 25 genera of cushion plants in Brassicaceae, within which six species within five genera occurred in China, i.e., *Alyssumklimesii* Al-Shehbaz (now *Ladakiellaklimesii* (Al-Shehbaz) D.A. German & Al-Shehbaz), *Ptilotrichumcanescens* (DC.) C.A. Mey (now *Steveniacanescens* (DC.) D.A. German), *Solms- laubachia eurycarpa* (Maxim.) Botsch., *Baimashaniapulvinata* Al-Shehbaz, *B.wangii* Al-Shehbaz, and *Shangrilaianana* Al-Shehbaz, J.P. Yue & H. Sun. Although many other Brassicaceae species were described as cushion plants and found to be occurring in China (Zhou et al. 2001; Al-Shehbaz 2015), they were not included in Aubert et al.’s catalogue (2014).

**Figure 1.**
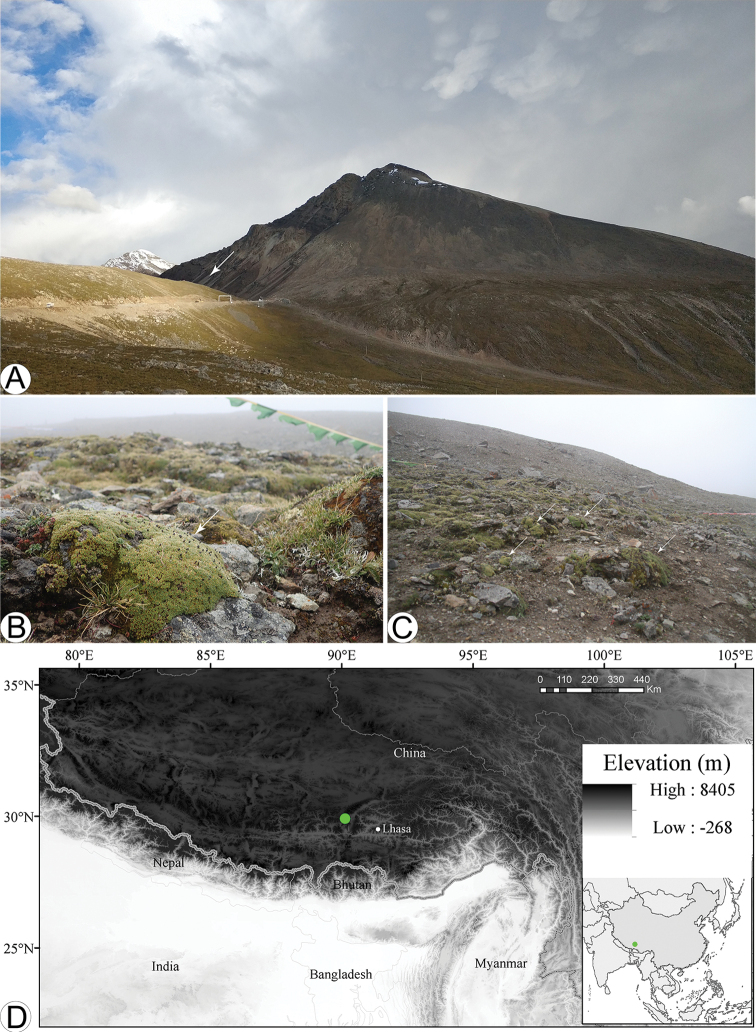
Habitat and geographic distribution of *Pulvinatusiaxuegulaensis***A–C** alpine meadow habitat, white arrow in **A** points to the location, white arrows in **B** and **C** point to *P.xuegulaensis***D** geographic distribution of *P.xuegulaensis*, marked with green circle. – Photos: **A** by Jianwen Zhang **B** and **C** by Lishen Qian.

**Figure 2.**
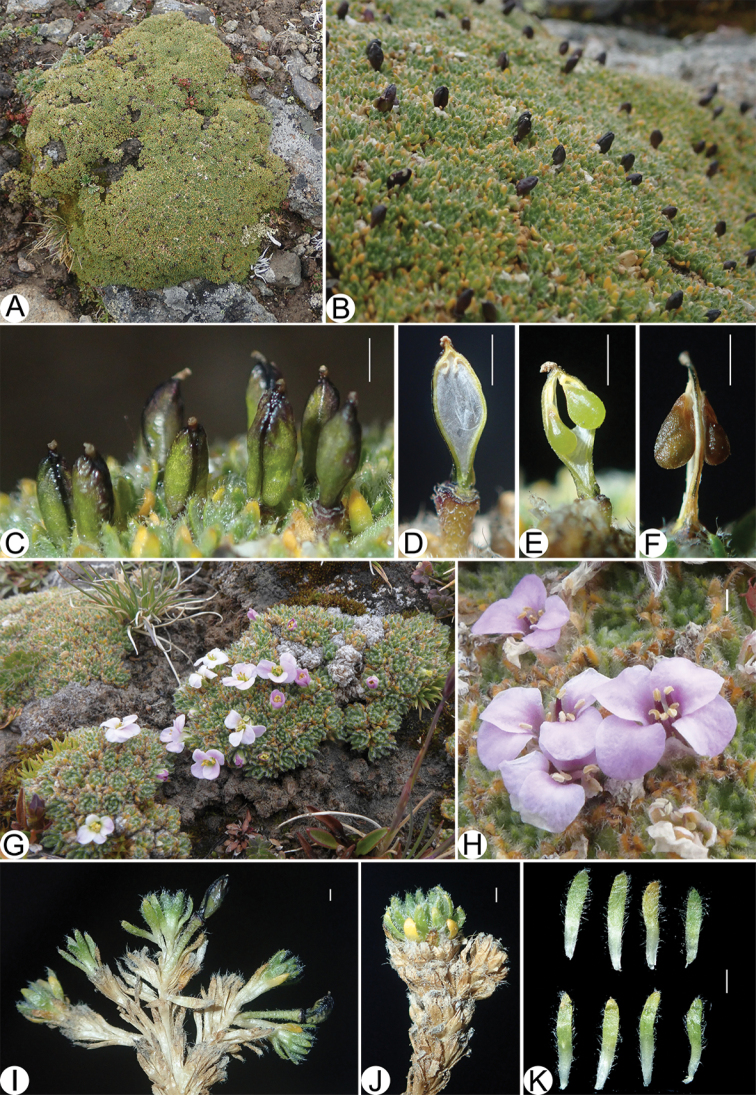
Images of *Pulvinatusiaxuegulaensis***A** and **B** fruiting plants **C** fruits **D** septum and replum **E** and **F** seeds **G** and **H** flowering plants **I** and **J** stems **K** leaves. Scales bars: 1 mm. – Photos: **A–F** & **I–K** by Lishen Qian **G** and **H** by Jianwen Zhang.

From 2000 to 2019, 58 new species of Brassicaceae from China were described (Du et al. 2020), the number of Chinese Brassicaceae species has grown to ca. 500 (Chen et al. 2019). During an expedition in August 2015 to Mt. Xuegu La, Damxung County, Xizang (Fig. 1), China, we collected a mustard plant with typical cushion characters and whitish pink flowers (Fig. 2G-H). We went back to the above-mentioned locality in August 2019 and collected fruiting material of this plant (Fig. 2A-F). Morphological studies family-wide revealed that it represents a new genus and species, hereafter recognized as *Pulvinatusiaxuegulaensis*. We also carried out molecular studies to verify its systematic position within the family.

## ﻿Material and methods

### ﻿Taxon sampling and data collection

To assess the identity and systematic position of the new taxon, a family-level sampling strategy was adopted. Two datasets, the nuclear ITS and plastomes, were utilized to reconstruct the phylogeny of Brassicaceae. The ITS dataset included 125 species representing 98 genera, of which two accessions of the novelty were newly sequenced. The plastome dataset included 74 species representing 70 genera, of which 16 accessions representing 16 species were newly sequenced. The plastome of *Bivonaealutea* (Biv.) DC. was extracted from raw sequencing data SRR8528386 deposited under NCBI BioProject PRJNA518905. *Cleomelutea* Hook. was chosen as outgroup for ITS and plastome datasets. Both ITS and plastome datasets comprised all 52 currently recognized tribes and nine genera which were not assigned to tribes within Brassicaceae. Data downloaded from GenBank and newly generated for this study are listed in Appendices 1 and 2, respectively.

### ﻿DNA extraction, amplification, and sequencing

Total genomic DNA was extracted from silica gel-dried fresh leaves using the Plant Genomic DNA Kit (Tiangen Biotech, Beijing, China) following the manufacturer’s protocol. The ITS region of one sample of *Pulvinatusiaxuegulaensis* (voucher specimens ZBFC-510) was amplified with the primers ITS-18F as modified by Mummenhoff et al. (1997) and ITS4 (White et al. 1990). A 25-ml polymerase chain reaction (PCR) included 1–2μL sample DNA (approx. 1–10 ng), 12.5μL Premix Taq TM (Takara Biomedical Technology, Beijing, China), 1μL of 10 μM stock of each primer, adjusted to 25 μL with ddH_2_O. The PCR program included a hot start with 4 min at 94 °C, and 30–32 cycles of amplification (1 min denaturing at 94 °C, 45–60 s annealing at 52–53 °C, 60–80 s extension at 72 °C), and a final elongation step for 10 min at 72 °C. The sequencing primers are the same as amplified primers. While the ITS region and plastome sequences of another sample of *P.xuegulaensis* (voucher specimens ZJW3454), together with the plastome data of 15 species listed in Appendix 2 were generated by genome skimming. Libraries for pair-end 150-bp sequencing was conducted using the Illumina HiSeq 2000 platform at Novogene Co. (Beijing, China).

### ﻿Data assembly and annotation

For the genome skimming data, low-quality reads were filtered, and the clean data were assembled using the GetOrganelle pipeline (Jin et al. 2020). The nuclear ITS and plastomes were also annotated using Geneious 8.2.4 (Kearse et al. 2012) with the published ITS of *C.himalaica* (AY662283) and plastome of *Rudolf*-*kamelinia korolkowii* (Regel & Schmalh.) Al-Shehbaz & D.A. German (KX886350) as the reference, respectively. Positions of start and stop codons of plastome sequences were checked manually.

### ﻿Sequence alignment and phylogenetic analyses

Two datasets, i.e., ITS and 75 plastid protein-coding genes (CDS) extracted from the annotated plastome sequences, were aligned using MAFFT v.7.311 (Katoh and Standley 2013) and manually adjusted with MEGA 7.0.14 (Kumar et al. 2016), ambiguous alignment regions within ITS dataset were trimmed by trimAl 1.2 (Capella-Gutiérrez et al. 2009). The 75 CDS were aligned one by one and then concatenated together, and substitutional saturation was assessed using DAMBE v.7.0.68 (Xia 2018).

Maximum parsimony (MP) and Bayesian Inference (BI) analyses were performed for the ITS dataset, while for the 75 CDS dataset, Maximum Likelihood (ML) method was utilized. No substitutional saturation was detected in 75 CDS dataset, as the index of substitution saturation (*Iss*) values were both significantly smaller than the critical *Iss (Iss.c)* values as defined by Xia et al. (2003). MP analysis was performed with heuristic searches of 1000 replicates with random stepwise addition using tree bisection reconnection (TBR) branch swapping as implemented in PAUP* 4.0a168 (Swofford 2020). All characters were weighted equally, and gaps were treated as missing data. BI and ML analyses were carried out with MrBayes v.3.2.6 (Ronquist et al. 2012) and RAxML 8.2.12 (Stamatakis 2014) implemented in the CIPRES Science Gateway v.3.3 (Miller et al. 2010), respectively. The best-fit model for nucleotide sequences was evaluated using jModeltest 2.1.6 (Darriba et al. 2012). Corrected Akaike Information Criterion (AICc) method was used to select the best-fit models. The SYM+I+G model were selected for ITS dataset in the BI analyses. Two independent runs each with four Monte Carlo Markov chains (MCMCs) were run for five million generations, and one tree sampled every 1000 generations. The first 1250 trees (25% of total trees) were discarded as burn-in. The remaining trees were summarized in a 50% majority-rule consensus tree, and the posterior probabilities (PP) were calculated. The ML analyses were conducted using the GTR+G model for 75 CDS dataset, with the option of rapid bootstrap of 1000 replicates.

## ﻿Results

### ﻿Morphological evaluation

With a single pivotal root, very short internode and compact branches, *Pulvinatusiaxuegulaensis* forms a hemispherical (dome) shape (Fig. 1B-C and Fig. 2A). Its leaves are linear-lanceolate and imbricate (Fig. 2I-K), and its fruits are ovoid silicles with stout fruit pedicles (Fig. 2C-F). These character combinations were not seen in any other Brassicaceae taxa occurring in China, suggesting it might represent a new species.

### ﻿Nuclear ITS and plastome assemblies

The ITS sequences for two accessions of the novelty were 628 bp long. Most of the 16 newly sequenced plastomes were assembled into complete circular genome, except one or two gaps remained in the noncoding regions of three accessions. Gaps information, voucher records, and GenBank accession numbers are provided in Appendix 2.

### ﻿Phylogenetic analysis

**Figure 3.**
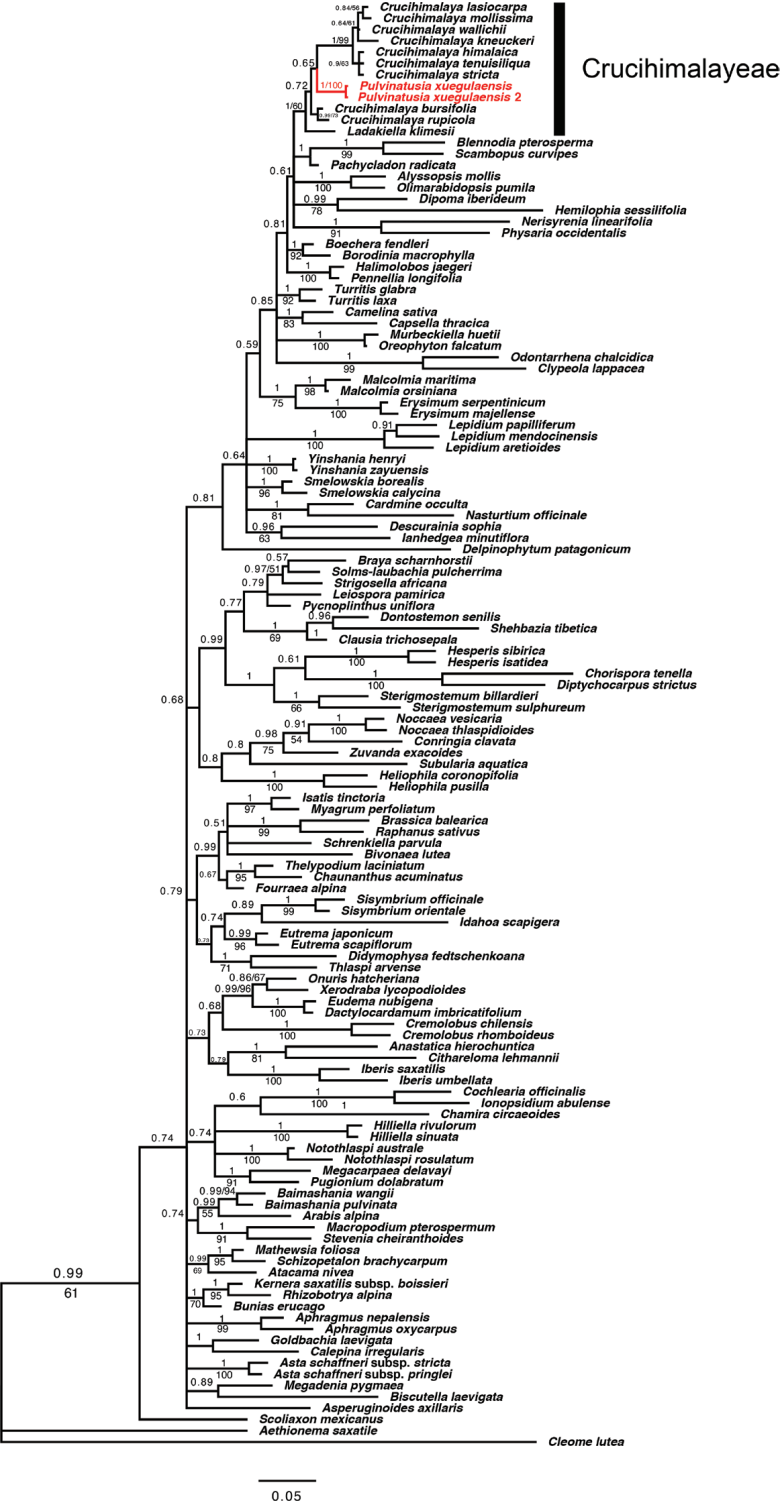
Bayesian Inference topology of the Brassicaceae relationships based on the nuclear ITS dataset. Bayesian inference posterior probability (PP) and maximum parsimony bootstrap (BS) are noted.

**Figure 4.**
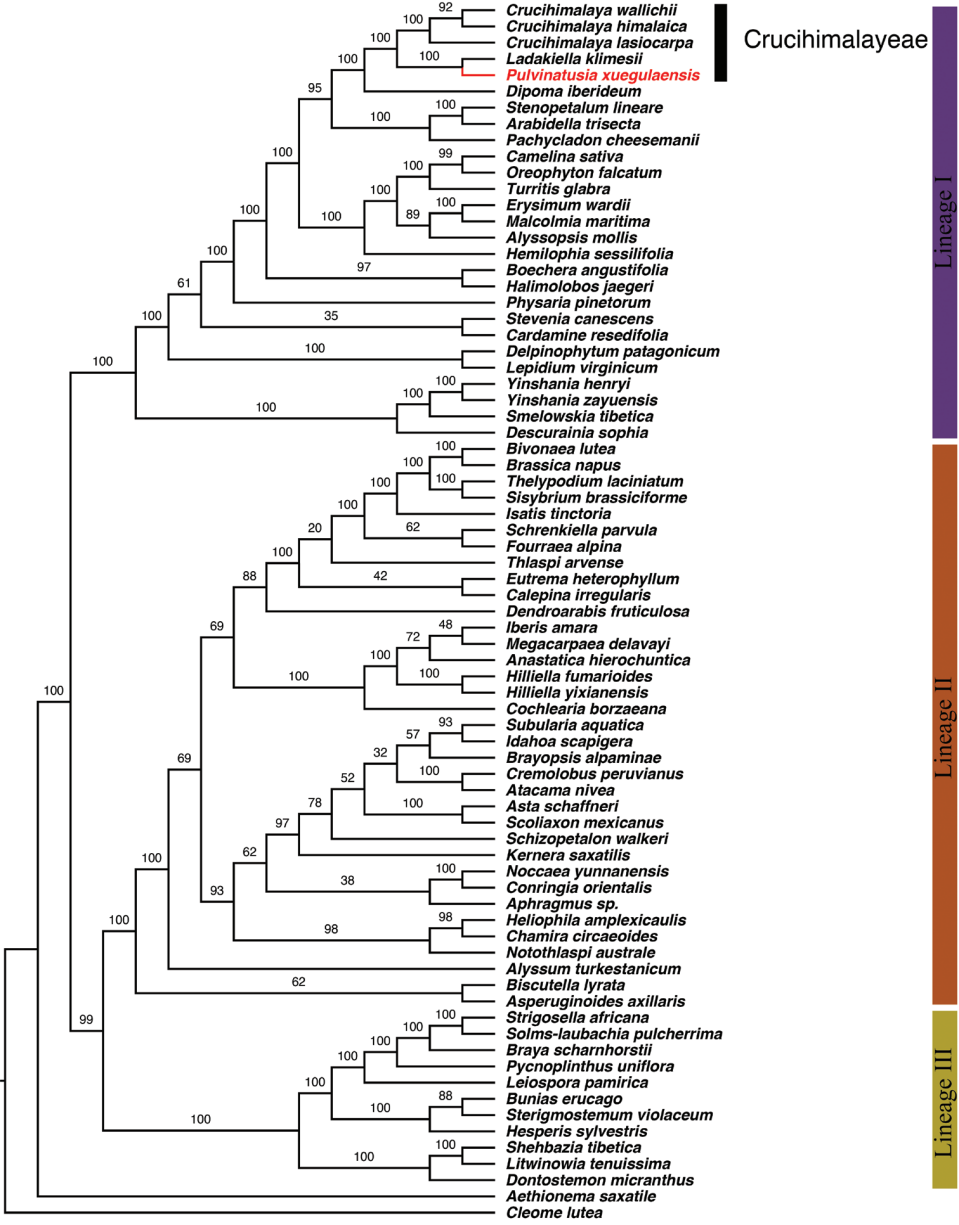
Maximum Likelihood cladogram of the Brassicaceae based on the plastome dataset. Maximum likelihood bootstraps (BS) are noted above the branch. Three Lineages of Brassicaceae (Beilstein et al. 2006; Walden et al. 2020) were marked.

The aligned ITS matrix was 496 bp long with 261 (52.6%) parsimony-informative sites. The aligned plastome CDS matrix was 61,713 bp long with 7,730 (12.5%) parsimony-informative sites. The resolution of MP analyses was relatively weaker than the outcome of BI analyses, thus only the topologies of Bayesian phylogenetic analysis were shown for ITS dataset. As our aim was to assess the systematic position of *Pulvinatusiaxuegulaensis*, only clades containing this taxon were concerned. In the ITS phylogeny, two accessions of *P.xuegulaensis* clustered together and embedded in a clade consisting of *Crucihimalaya* species. This *P.xuegulaensis/Crucihimalaya* clade is sister to *Ladakiellaklimesii* (Fig. 3). In the plastome phylogeny (Fig. 4), only three *Crucihimalaya* species and one accession for each of *P.xuegulaensis* and *L.klimesii* were sampled. The sequence of *P.xuegulaensis* formed a clade with *L.klimesii*, and then sistered to a clade composed of three *Crucihimalaya* species. Therefore, both nuclear and chloroplast phylogenies indicated that *P.xuegulaensis* should be assigned to the tribe Crucihimalayeae.

### ﻿Taxonomic treatment

#### ﻿ Pulvinatusia

Taxon classificationPlantaeBrassicalesBrassicaceae

J.P. Yue, H.L. Chen, Al-Shehbaz & H. Sun gen. nov.

urn:lsid:ipni.org:names:77249032-1

Fig. 2

##### Type.

*Pulvinatusiaxuegulaensis* J.P. Yue, H.L. Chen, Al-Shehbaz & H. Sun.

##### Diagnosis.

As indicated above, the monospecific *Pulvinatusiaxuegulaensis* and *Ladakiellaklimesii* are the only members of the tribe Crucihimalayeae with pulvinate and scapose habit and pink to whitish pink petals. The former differs by having simple and fewer forked trichomes, thin papery leaves, solitary flowers, caducous sepals, and glabrous, somewhat flattened fruits. By contrast, *L.klimesii* has subdendritic trichomes with finely branched rays, thick and fleshy leaves, 2–4-flowered racemes, persistent sepals, and pubescent and terete fruits.

##### Description.

Herbs perennial, cespitose, scapose, pulvinate, with well-developed caudex covered with petioles of previous years. Trichomes simple, mixed with fewer forked stalked ones. Leaves densely imbricate, sessile, thin, papery, densely long ciliate, midvein obscure, adaxially concave to nearly flat, base attenuate, apex subacute. Flowers solitary on short pedicels originating from axils of basal leaves. Fruiting pedicels stout, erect or ascending, often hidden among basal leaves. Sepals oblong, abaxially with trichomes similar to those on leaves. Petals whitish pink to pink; blade obovate to suborbicular, apex obtuse, rounded or rarely acute, claw subequaling or slightly shorter than sepals. Stamens 6, slightly tetradynamous; filaments unappendaged, free; anthers ovate or oblong, obtuse at apex. Ovules 2 or 3 per ovary, placentation parietal. Fruits dehiscent, latiseptate, ovoid to ellipsoid, inflated; valves thick leathery, carinate; replum rounded, visible; septum complete; style obsolete or short and to 0.4 mm long, stout; stigma capitate, entire, unappendaged. Seeds aseriate, wingless, oblong, seed coat smooth, not mucilaginous when wetted; cotyledons accumbent.

##### Name derivation.

The generic name is derived from the pulvinate habit of the plant, and the species epithet from the Xuegu La (Xizang, China), where the type collection was made.

#### ﻿ Pulvinatusia xuegulaensis

Taxon classificationPlantaeBrassicalesBrassicaceae

J. P. Yue, H. L. Chen, Al-Shehbaz & H. Sun sp. nov.

urn:lsid:ipni.org:names:77249034-1

Fig. 2

##### Description.

Herbs 0.9–1.6 cm tall; caudex many branched, to 4 mm in diam. Trichomes simple, to 0.6 mm long, mixed with fewer forked stalked ones. Leaves densely imbricate, (3.5–) 4.0–4.2 (–4.7) × 0.7–1 mm, thin, papery, long ciliate on both sides and margin. Flowers solitary on short pedicels originating from axils of basal leaves. Fruiting pedicels stout, 4.5–6 mm long, often hidden among basal leaves. Sepals oblong, 1–2 × ca. 1 mm. Petals whitish pink or pink; obovate to suborbicular, 3–3.5 × 2.5–3 mm, claw 2.5–3.4 mm long. Filaments 1.5–2 mm long; anthers 0.3–0.5 mm long. Ovules 2 or 3 per ovary. Fruit ovoid to ellipsoid, 1.6–1.9 × 0.8–1 mm; valves thick leathery, inflated, style 0.2–0.4 mm long. Seeds 1–1.5 × 0.7–0.9 mm, seed coat smooth, not mucilaginous when wetted; cotyledons accumbent.

##### Type.

China. Xizang: Xuegu La, alpine meadow, sandy area, 29°55' N, 90°7' E, 5300 m, 4 Aug. 2019, ZBFC-510 (holotype, KUN!; isotype, KUN!). ***Paratype*.** China. Xizang: Xuegu La, alpine gravel slopes, sandy area, 29°54' N, 90°7' E, 5407 m, 28 Aug. 2015, ZJW3454 (KUN).

## ﻿Discussion

*Pulvinatusiaxuegulaensis* displays typical cushion-plants morphology, which belongs to the dome type of Aubert et al.’s category (2014). Many ball-shaped individuals grow together along alpine slopes and form a community with spectacular landscape (Fig. 1B-C). Without flowers and fruits, one can easily misidentify *P.xuegulaensis* as *Arenariabryophylla* Fernald, a member of Caryophyllaceae family and one of the most typical cushion plants in the Sino-Himalayas. This might partially explain why this new taxon remained unrecognized until now; even the type locality is nearby a county road (Fig. 1A). Only with its conspicuous cruciform pink flowers and ovoid silicles, one can easily recognize it as Brassicaceae. To date, only one population of *P.xuegulaensis* has been found, within the family and the six cushion taxa (as mentioned in the Introduction) listed by Aubert et al. (2014) occurring in China, *P.xuegulaensis* is most similar to *Ladakiellaklimesii* in gross morphology. Whereas it differs from the latter by more (vs. less) compact branches; imbricate (vs. rosulate) leaves; solitary flowers (vs. 2–4-flowered raceme) and stout (vs. slender) fruiting pedicel. By contrast, these distinct characters of *P.xuegulaensis* are also shown in *Xerodrabapatagonica* (Speg.) Skottsb. (Eudemeae, Brassicaceae) (Table 1), a South American species endemic to southern Argentina and Chile at an altitude of 20 – 1050 m (Salariato et al. 2015a), demonstrating morphological homoplasy between unrelated taxa of different continents.

**Table 1.** Tribal assignments and comparisons of morphological characters of *Pulvinatusiaxuegulaensis*, *Ladakiellaklimesii* and *Xerodrabapatagonica.*

	*Pulvinatusiaxuegulaensis*	*Ladakiellaklimesii*	*Xerodrabapatagonica*
Tribal assignments	Crucihimalayeae	Crucihimalayeae	Eudemeae
Habit	perennial, pulvinate	perennial, pulvinate	perennial, pulvinate
Type of cushions	hemispherical (dome shaped) cushion	hemispherical (dome shaped) cushion	low cushion
Compactness	compact, hard	intermediate	compact, hard
Leaf arrangement	imbricate	rosulate	imbricate
Leaf shape	linear-lanceolate	obovate to spatulate	oblong-ovate
Leaf texture	thin, papery	thickened, fleshy	thickened, fleshy
Flower	solitary	raceme 2–4-flowered	solitary
Petal color	whitish pink, pink	pink throughout or white with pink claws	white to pale yellow
Fruit	ovoid to ellipsoid silicle	ovoid silicle	ellipsoid silicle
Fruiting pedicels	stout	slender	slender
Fruit valves	thick leathery, carinate, glabrous	papery, not veined, densely tomentose outside	leathery, carinate, glabrous

In both nuclear and chloroplast phylogenies, *Pulvinatusiaxuegulaensis* fell in a clade consisting of *Ladakiella* and *Crucihimalaya* species, indicating that the new taxon is phylogenetically close to these two genera, which had been assigned to the tribe Crucihimalayeae by German and Al-Shehbaz (2010). This study therefore supported *Pulvinatusia* to be the third genus within Crucihimalayeae. However, the intergeneric relationship within this tribe was not resolved. In the nuclear rDNA (ITS) phylogeny, two accessions of *P.xuegulaensis* were embedded in a clade consisting of nine *Crucihimalaya* species and then sister to *L.klimesii* (Fig. 3). This indicates that the genus *Crucihimalaya* as currently delimited (German 2005; Al-Shehbaz et al. 2011) is not monophyletic. In fact, generic delimitation and systematic position of *Crucihimalaya* have been in dispute for a long time. This genus was first established by Al-Shehbaz et al. (1999) to accommodate nine species excluded from *Arabidopsis* based on morphological and molecular evidences (Price et al. 1994; O’Kane et al. 1995). This delimitation was followed by Zhou et al. (2001) and Appel and Al-Shehbaz (2003), and the genus had been assigned to the tribe Camelineae by Al-Shehbaz et al. (2006) in their first scheme of tribal classification. However, subsequent molecular studies revealed that *Crucihimalaya* is phylogenetically distant to taxa from Camelineae but formed a clade with species *Arabistibetica* Hook.f. & Thomson, *A.tenuisiliqua* Rech.f. & Köie, *A.rupicola* Krylov, *Transberingiabursifolia* (DC.) Al-Shehbaz & O’Kane and *Alyssumklimesii* (O’kane and Al-Shehbaz 2003; Koch et al. 2007; Warwick et al. 2008; German et al. 2009). These species then had been transferred to *Crucihimalaya* and resulted in a heterogeneous genus including 13 species (German and Ebel 2005; German 2005; Al-Shehbaz et al. 2011), whereas a new genus *Ladakiella* was created to accommodate *L.klimesii* excluded from *Alyssum* (German and Al-Shehbaz 2010). Both *Ladakiella* and *Crucihimalaya* s.l. were assigned to the newly proposed tribe Crucihimalayeae (German and Al-Shehbaz 2010). The ITS phylogeny constructed in this study suggested either to combine *P.xuegulaensis* with *Crucihimalaya* s.l. or split the latter genus into several segregates. *Pulvinatusiaxuegulaensis* is very similar to *L.klimesii* in gross morphology as they both share pulvinate habit and inflated ovoid silicles. These morphological similarities corresponded to their phylogenetic relationships revealed in the plastome phylogeny, within which these two species formed a clade sister to three *Crucihimalaya* species (Fig. 4). The discrepancy between nuclear and chloroplast phylogenies revealed in this study might be attributed to two main reasons: 1) sampling difference, i.e., there are nine species from *Crucihimalaya* s.l. sampled in the ITS phylogeny, but only three species sampled in the plastome phylogeny, especially lack of *C.bursifolia* and *C.rupicola*. 2) reticulate evolution caused by hybridization and/or introgression, of which evolutionary processes have been proposed for numerous taxa in the mustard family (Mummenhoff et al. 2004; Lihová et al. 2006; Dierschke et al. 2009; German and Friesen 2014; Mandáková et al. 2017; Hohmann and Koch 2017; Chen et al. 2020). To clarify inter- and infrageneric relationships within Crucihimalayeae, studies with comprehensive sampling and more molecular markers are needed.

The discovery of *Pulvinatusiaxuegulaensis* added one new genus and species to the cushion plant list compiled by Aubert et al. (2014). The cushion habit had long been considered a good example of evolutionary convergence among various plants in alpine and arctic regions (Aubert et al. 2014). It had been suggested to evolve independently four times in South American Brassicaceae (Salariato et al. 2015b) and happened at least 115 times in whole angiosperms (Boucher et al. 2016). Characterized by dense branches and compact structure, cushion plants usually form hemispheric or mat shapes, which enables them to adapt to cold and/or dry harsh environments and also facilitate other alpine plant species by nurse trait effects (Körner 2003; Yang et al. 2010; Chen et al. 2015; Chen et al. 2017; Yang et al. 2017). However, nothing is known about the underlying genetic basis of adaptation to alpine environments of cushion plants. All the three genera of Crucihimalayeae coexist in Qinghai-Tibet Plateau, and all species of *Crucihimalaya* are not pulvinate, while both *L.klimesii* and *P.xuegulaensis* are cushion species, thus provide an excellent system to decode the genetic basis of the formation of cushion structure and study the adaptive evolution of cushion plants, and the available genome of *C.himalaica* (Zhang et al. 2019) can facilitate this process.

## Supplementary Material

XML Treatment for Pulvinatusia

XML Treatment for Pulvinatusia xuegulaensis

## ﻿Appendix 1

**Taxon and GenBank accession numbers for the ITS and plastid genome sequences downloaded from GenBank and used in this study.**

**ITS:Outgroup**: ***Cleomelutea*** Hook. (AF137588); **Ingroups: Aethionemeae: *Aethionemasaxatile*** (L.) W.T. Aiton (GQ284853), **Alysseae**: ***Clypeolalappacea*** Boiss. (EF514645), ***Odontarrhenachalcidica*** (Janka) Španiel & al. (GQ284877), **Alyssopsideae: *Alyssopsismollis*** (Jacq.) O. E. Schulz (GQ424523), ***Olimarabidopsispumila*** (Stephan) Al-Shehbaz, O’Kane & R. A. Price (AY662277), **Anastaticeae: *Anastaticahierochuntica*** L. (GQ424524), ***Citharelomalehmannii*** Bunge (DQ357528), **Anchonieae: *Sterigmostemumbillardieri*** (DC.) D.A. German (DQ357512), ***Sterigmostemumsulphureum*** (Banks & Sol.) Bornm. (KJ663764), **Aphragmeae: *Aphragmusnepalensis*** (H. Hara) Al-Shehbaz (DQ165335), ***Aphragmusoxycarpus*** (Hook. f. & Thomson) Jafri (DQ165337), **Arabideae: *Arabisalpina*** L. (DQ060111), ***Baimashaniapulvinata*** Al-Shehbaz (FJ187969), ***Baimashaniawangii*** Al-Shehbaz (JQ919842), **Asteae: Astaschaffnerisubsp.pringlei** (O.E. Schulz) Al-Shehbaz (HQ541169), **Astaschaffnerisubsp.stricta** (Rollins) Al-Shehbaz (HQ541171), **Biscutelleae: *Biscutellalaevigata*** L. (KF022694), ***Megadeniapygmaea*** Maxim. (KX943555), **Bivonaeeae**: ***Bivonaealutea*** (Biv.) DC. (HQ327490), **Boechereae**: ***Boecherafendleri*** (S. Watson) W. A. Weber (JX146958), ***Borodiniamacrophylla*** (Turcz.) O. E. Schulz (EU274865), **Brassiceae**: ***Brassicabalearica*** Pers. (AF263402), ***Raphanussativus*** L. (FJ980407), **Buniadeae**: ***Buniaserucago*** L. (GQ497885), **Calepineae**: ***Calepinairregularis*** (Asso) Thell. (DQ249822), ***Goldbachialaevigata*** (M. Bieb.) DC. (DQ357546), **Camelineae**: ***Camelinasativa*** (L.) Crantz (KJ623504), ***Capsellathracica*** Velen. (HE575243), **Cardamineae**: ***Cardamineocculta*** Hornem. (KX244391), ***Nasturtiumofficinale*** W. T. Aiton (AY254531), **Chorisporeae**: ***Chorisporatenella*** (Pall.) DC. (DQ249866), ***Diptychocarpusstrictus*** (Fisch. ex M. Bieb.) Trautv. (DQ357534), **Cochlearieae**: ***Cochleariaofficinalis*** L. (HQ268642), ***Ionopsidiumabulense*** (Pau) Rothm. (HQ268661), **Coluteocarpeae**: ***Noccaeathlaspidioides*** (Pall.) F. K. Mey. (DQ249838), ***Noccaeavesicaria*** (L.) Al-Shehbaz (GQ497857), **Conringieae**: ***Conringiaclavata*** Boiss. (AY722505), ***Zuvandaexacoides*** (DC.) Askerova (DQ357607), **Cremolobeae**: ***Cremolobuschilensis*** (Lag. ex DC.) DC. (GQ424530), ***Cremolobusrhomboideus*** Hook. (KF662762), **Crucihimalayeae**: ***Crucihimalayabursifolia*** (DC.) D. A. German & A. L. Ebel (AF137557), ***Crucihimalayahimalaica*** (Edgew.) Al-Shehbaz, O’Kane & R. A. Price (AY662283), ***Crucihimalayakneuckeri*** (Bornm.) Al-Shehbaz, O’Kane & R. A. Price (AF137550), ***Crucihimalayamollissima*** (C.A. Mey.) Al-Shehbaz, O’Kane & R. A. Price (DQ249845), ***Crucihimalayalasiocarpa*** (Hook.f. & Thomson) Al-Shehbaz, O’Kane & R. A. Price (AF137556), ***Crucihimalayarupicola*** (Krylov) A. L. Ebel & D. A. German (FJ187923), ***Crucihimalayastricta*** (Cambess.) Al-Shehbaz, O’Kane & R. A. Price (AF137554), ***Crucihimalayatenuisiliqua*** (Rech.f. & Köie) Al-Shehbaz, D. A. German & M. A. Koch (KF547304), ***Crucihimalayawallichii*** (Hook.f. & Thomson) Al-Shehbaz, O’Kane & R. A. Price (AY662282), ***Ladakiellaklimesii*** (Al-Shehbaz) D. A. German & Al-Shehbaz (EF514608), **Descurainieae**: ***Descurainiasophia*** (L.) Webb ex Prantl (AF205587), ***Ianhedgeaminutiflora*** (Hook.f. & Thomson) Al-Shehbaz & O’Kane (HQ896625), **Dontostemoneae**: ***Clausiatrichosepala*** (Turcz.) F. Dvořák (LK021263), ***Dontostemonsenilis*** Maxim. (LK021244), **Erysimeae**: ***Erysimummajellense*** Polatschek (KJ418042); ***Erysimumserpentinicum*** Polatschek (KJ418068), **Euclidieae**: ***Brayascharnhorstii*** Regel & Schmalh. (MH23787), ***Leiosporapamirica*** (Botsch. & Vved.) Botsch. & Pachom. (MH237698), ***Pycnoplinthusuniflora*** (Hook. f. & Thomson) O. E. Schulz (MH237701), ***Solms*-*laubachia pulcherrima*** Muschl. (MH237723), ***Strigosellaafricana*** (L.) Botsch. (MH237728), **Eudemeae**: ***Dactylocardamumimbricatifolium*** Al-Shehbaz (KM376257), ***Eudemanubigena*** Humb. & Bonpl. (KC174370), ***Onurishatcheriana*** (Gilg ex Macloskie) Gilg & Muschl. (KM376239), ***Xerodrabalycopodioides*** (Speg.) Skottsb. (KM376221), **Eutremeae**: ***Eutremajaponicum*** (Miq.) Koidz. (JN387782), ***Eutremascapiflorum*** (Hook. f. & Thomson) Al-Shehbaz, G. Q. Hao & J. Q. Liu (DQ518398), **Halimolobeae**: ***Halimolobosjaegeri*** (Munz) Rollins (AF137567), ***Pennellialongifolia*** (Benth.) Rollins (AF307627), **Heliophileae**: ***Heliophilacoronopifolia*** L. (DQ249846), ***Heliophilapusilla*** L. f. (LN589686), **Hesperideae**: ***Hesperissibirica*** L. (DQ357549), ***Hesperisisatidea*** (Boiss.) D.A. German & Al-Shehbaz (GQ497882), **Hillielleae**: ***Hilliellarivulorum*** (Dunn) Y. H. Zhang & H. W. Li (KX244376), ***Hilliellasinuata*** (K.C. Kuan) Y. H. Zhang & H. W. Li (KX244377), **Iberideae**: ***Iberissaxatilis*** L. (LN589689), ***Iberisumbellata*** L. (AY237921), **Isatideae**: ***Isatistinctoria*** L. (GQ131323), ***Myagrumperfoliatum*** L. (GQ424547), **Kernereae**: **Kernerasaxatilissubsp.boissieri** (Reut.) Nyman (AJ440314), ***Rhizobotryaalpina*** Tausch (AJ440315), **Lepidieae**: ***Delpinophytumpatagonicum*** (Speg.) Speg. (KM376225), ***Lepidiumaretioides*** (Hedge) Al-Shehbaz (GQ497859), ***Lepidiumpapilliferum*** (L.F. Hend.) A. Nelson & J.F. Macbr. (JF541495), ***Lepidiummendocinensis*** (Hauman) Al-Shehbaz (GQ497890), **Malcolmieae**: ***Malcolmiamaritima*** (L.) W. T. Aiton (AM905723), ***Malcolmiaorsiniana*** (Ten.) Ten. (DQ357560), **Megacarpaeeae**: ***Megacarpaeadelavayi*** Franch. (KX244385), ***Pugioniumdolabratum*** Maxim. (JF978171), **Microlepidieae**: ***Blennodiapterosperma*** (J.M. Black) J. M. Black (DQ357519), ***Pachycladonradicata*** (Hook. f.) Heenan & A. D. Mitch. (EF015693), ***Scambopuscurvipes*** (F. Muell.) O. E. Schulz (JX630167), **Notothlaspideae**: ***Notothlaspiaustrale*** Hook. f. (AF100689), ***Notothlaspirosulatum*** Hook. f. (AF100690), **Oreophytoneae**: ***Murbeckiellahuetii*** (Boiss.) Rothm. (GQ424546), ***Oreophytonfalcatum*** (E. Fourn.) O. E. Schulz (GQ424549), **Physarieae**: ***Nerisyrenialinearifolia*** (S. Watson) Greene (AF055200), ***Physariaoccidentalis*** (S. Watson) O’Kane & Al-Shehbaz (KU975797), **Schizopetaleae**: ***Mathewsiafoliosa*** Hook. & Arn. (KC174387), ***Schizopetalonbrachycarpum*** Al-Shehbaz (KC174407), **Scoliaxoneae**: ***Scoliaxonmexicanus*** (S. Watson) Payson (HQ541175), **Shehbazieae**: ***Shehbaziatibetica*** (Maxim.) D. A. German (LN713855), **Sisymbrieae**: ***Sisymbriumofficinale*** (L.) Scop. (AF531565), ***Sisymbriumorientale*** L. (AF531592), **Smelowskieae**: ***Smelowskiaborealis*** (Greene) W. H. Drury & Rollins (AY230571), ***Smelowskiacalycina*** (Stephan ex Willd.) C. A. Mey. (AY230604), **Stevenieae**: ***Macropodiumpterospermum*** F. Schmidt (GU182055), ***Steveniacheiranthoides*** DC. (GU182059), **Thelypodieae**: ***Chaunanthusacuminatus*** (Rollins) R. A. Price & Al-Shehbaz (GQ497855), ***Thelypodiumlaciniatum*** (Hook.) Endl. (KJ953749), **Thlaspideae**: ***Didymophysafedtschenkoana*** Regel (EF514647), ***Thlaspiarvense*** L. (KT220620), **Turritideae**: ***Turritisglabra*** L. (DQ518389), ***Turritislaxa*** (Sm.) Hayek (KF547126), **Yinshanieae**: ***Yinshaniahenryi*** (Oliv.) Y. H. Zhang (KX244390), ***Yinshaniazayuensis*** Y. H. Zhang (KX244395), **Unassigned genera:*Asperuginoidesaxillaris*** (Boiss. & Hohen.) Rauschert (EF514626), ***Atacamanivea*** (Phil.) Toro, Mort & Al-Shehbaz (KC174381), ***Chamiracircaeoides*** (L.f.) Zahlbr. (AJ862719, AJ862720), ***Dipomaiberideum*** Franch. (GQ497861), ***Fourraeaalpina*** (L.) Greuter & Burdet (DQ518395), ***Hemilophiasessilifolia*** Al-Shehbaz, Arai & H. Ohba (KT762595), ***Idahoascapigera*** (Hook.) A. Nelson & J. F. Macbr. (MF964066), ***Schrenkiellaparvula*** (Schrenk) D. A. German & Al-Shehbaz (AF137579), ***Subulariaaquatica*** L. (MF963829).

**Plastid genome**: **Outgroup**: ***Cleomelutea*** Hook. (MK637687), **Ingroups**: **Aethionemeae**: ***Aethionemasaxatile*** (L.) W. T. Aiton (MK637661), **Alysseae**: ***Alyssumturkestanicum*** Regel & Schmalh. (KY498535), **Alyssopsideae**: ***Alyssopsismollis*** (Jacq.) O. E. Schulz (MK637657), **Anastaticeae**: ***Anastaticahierochuntica*** L. (KY912021), **Anchonieae**: ***Sterigmostemumviolaceum*** (Botsch.) H. L. Yang (MK637808), **Asteae**: ***Astaschaffneri*** (S. Watson) O. E. Schulz (MK637662), **Biscutelleae**: ***Biscutellalyrata*** L. (MH359179), **Bivonaeeae**: ***Bivonaealutea*** (Biv.) DC. (SRR8528386), **Boechereae**: ***Boecheraangustifolia*** (Nutt.) Dorn (MK637673), **Brassiceae**: ***Brassicanapus*** L. (GQ861354), **Buniadeae**: ***Buniaserucago*** L. (LN877377), **Calepineae**: ***Calepinairregularis*** (Asso) Thell. (MK637682), **Camelineae**: ***Camelinasativa*** (L.) Crantz (LN877386), **Cardamineae**: ***Cardamineresedifolia*** L. (KJ136822), **Chorisporeae**: ***Litwinowiatenuissima*** (Pall.) Woronow ex Pavlov (MK637744), **Cochlearieae**: ***Cochleariaborzaeana*** (Coman & Nyár.) Pobed. (LN866844), **Conringieae**: ***Conringiaorientalis*** (L.) C. Presl (MK637689), **Cremolobeae: *Cremolobusperuvianus*** (Lam.) DC. (MK637692), **Crucihimalayeae**: ***Crucihimalayalasiocarpa*** (Hook. f. & Thomson) Al-Shehbaz, O’Kane & R. A. Price (MK637686), ***Crucihimalayawallichii*** (Hook. f. & Thomson) Al-Shehbaz, O’Kane & R. A. Price (AP009372), ***Ladakiellaklimesii*** (Al-Shehbaz) D. A. German & Al-Shehbaz (MK637741), **Dontostemoneae**: ***Dontostemonmicranthus*** C. A. Mey. (KY912023), **Euclidieae**: ***Brayascharnhorstii*** Regel & Schmalh. (MT845129), ***Leiosporapamirica*** (Botsch. & Vved.) Botsch. & Pachom. (MT845148), ***Pycnoplinthusuniflora*** (Hook. f. & Thomson) O. E. Schulz (MT845156), ***Strigosellaafricana*** (L.) Botsch. (MT845193), **Eudemeae**: ***Brayopsisalpaminae*** Gilg & Muschl. (MK637666), ***Solms*-*laubachia pulcherrima*** Muschl. (MT845182), **Eutremeae**: ***Eutremaheterophyllum*** (W. W. Sm.) H. Hara (KT270358), **Halimolobeae**: ***Halimolobosjaegeri*** (Munz) Rollins (MK637824), **Heliophileae**: ***Heliophilaamplexicaulis*** L. f. (MK637720), **Hesperideae**: ***Hesperissylvestris*** Crantz (KY912027), **Iberideae**: ***Iberisamara*** L. (MK637733), **Isatideae**: ***Isatistinctoria*** L. (KT591187), **Kernereae**: ***Kernerasaxatilis*** (L.) Sweet (MK637737), **Lepidieae**: ***Delpinophytumpatagonicum*** (Speg.) Speg. (K637706), ***Lepidiumvirginicum*** L. (AP009374), **Malcolmieae**: ***Malcolmiamaritima*** (L.) W. T. Aiton (MK637751), **Megacarpaeeae**: ***Megacarpaeadelavayi*** Franch. (KX886349), **Microlepidieae**: ***Arabidellatrisecta*** (F. Muell.) O. E. Schulz (MK637664), ***Pachycladoncheesmanii*** Heenan & A. D. Mitch. (JQ806762), ***Stenopetalumlineare*** R. Br. ex DC. (MK637800), **Notothlaspideae**: ***Notothlaspiaustrale*** Hook. f. (MK637761), **Oreophytoneae**: ***Oreophytonfalcatum*** (E. Fourn.) O. E. Schulz (MK637767), **Physarieae**: ***Physariapinetorum*** (Wooton & Standl.) O’Kane & Al-Shehbaz (MK637778), **Schizopetaleae**: ***Schizopetalonwalkeri*** Sims (MK637809), **Scoliaxoneae**: ***Scoliaxonmexicanus*** (S. Watson) Payson (MK637801), **Shehbazieae**: ***Shehbaziatibetica*** (Maxim.) D. A. German (MK637829), **Thelypodieae**: ***Thelypodiumlaciniatum*** (Hook.) Endl. (MK637813), **Thlaspideae**: ***Thlaspiarvense*** L. (KX886351), **Unassigned genera:*Atacamanivea*** (Phil.) Toro, Mort & Al-Shehbaz (MK637821), ***Chamiracircaeoides*** (L. f.) Zahlbr. (MK637678), ***Dipomaiberideum*** Franch. (MK637702), ***Fourraeaalpina*** (L.) Greuter & Burdet (MK637717), ***Hemilophiasessilifolia*** Al-Shehbaz, Arai & H. Ohba (MK637730), ***Idahoascapigera*** (Hook.) A. Nelson & J. F. Macbr. (MK637735), ***Schrenkiellaparvula*** (Schrenk) D. A. German & Al-Shehbaz (KT222186), ***Subulariaaquatica*** L. (MK637792).

## ﻿Appendix 2

**Table:** Species and data description of ITS and plastomes used in this study.

Taxon	Voucher Specimens	Locations	ITS GenBank numbers	Plastome GenBank numbers	Plastome length (bp)	Number of plastome gap
*Aphragmus sp.*	YZC250 (KUN)	Daocheng, China		OL800589	153022	
*Asperuginoidesaxillaris*	SunHang17434 (KUN)	Uzbekistan		OL800590	153193	
*Crucihimalayahimalaica*	No specimen	Batang, China		OL800599	155112	
*Dendroarabisfruticulosa*	YC-XZ019 (KUN)	Altay, China		OL800587	152755	
*Descurainiasophia*	YC-XZ070 (KUN)	Fukang, China		OL800591	153829	
*Erysimumwardii*	YZC202 (KUN)	Lhasa, China		OL800596	154466	
*Hilliellafumarioides*	ZJW4302 (KUN)	Jinhua, China		OL800598	154988	
*Hilliellayixianensis*	ZJW4330 (KUN)	Yixian, China		OL800594	154334	2 gaps
*Noccaeayunnanensis*	YZC223 (KUN)	Shangrila, China		OL800588	152801	
*Pulvinatusiaxuegulaensis*	ZBFC-510 (KUN)	Damxung, China	OL828562			
*P.xuegulaensis*	ZJW3454 (KUN)	Damxung, China	OL828563	OL800600	155134	
*Sisymbriumbrassiciforme*	YC-XZ025 (KUN)	Altay, China		OL800593	154238	
*Smelowskiatibetica*	YC-XZ132 (KUN)	Rutog, China		OL800595	154433	
*Steveniacanescens*	YC-XZ140 (KUN)	Gar, China		OL800597	154667	
*Turritisglabra*	YC-XZ035 (KUN)	Burqin, China		OL800592	154196	1 gap
*Yinshaniahenryi*	ZJW4523 (KUN)	Nanchuan, China		OL800602	155553	1 gap
*Yinshaniazayuensis*	ZJW4430 (KUN)	Zhangjiajie, China		OL800601	155401	
